# The effect of pre-laying maternal immunization on offspring growth and immunity differs across experimentally altered postnatal rearing conditions in a wild songbird

**DOI:** 10.1186/s12983-018-0272-y

**Published:** 2018-06-19

**Authors:** Rafał Martyka, Ewa B. Śliwińska, Mirosław Martyka, Mariusz Cichoń, Piotr Tryjanowski

**Affiliations:** 1 Institute of Nature Conservation, Polish Academy of Sciences, Mickiewicza 33, 31-120 Kraków, Poland; 2 Institute of Environmental Sciences, Jagiellonian University, Gronostajowa 7, 30-387 Kraków, Poland; 3 Institute of Zoology, Poznań University of Life Sciences, Wojska Polskiego 71C, 60-625 Poznań, Poland

**Keywords:** Brood size manipulation, Food availability, Great tit, Humoral immune response, LPS, Maternal antibodies, *Parus major*, Prenatal maternal effects

## Abstract

**Background:**

Prenatal antibody transfer is an immune-mediated maternal effect by which females can shape postnatal offspring resistance to pathogens and parasites. Maternal antibodies passed on to offspring provide primary protection to neonates against diverse pathogenic antigens, but they may also affect offspring growth and influence the development of an offspring’s own immune response. The effects of maternal antibodies on offspring performance commonly require that the disease environment experienced by a mother prior to breeding matches the environment encountered by her offspring after hatching/birth. However, other circumstances, like postnatal rearing conditions that affect offspring food availability, may also determine the effects of maternal antibodies on offspring growth and immunity. To date, knowledge about how prenatal immune-mediated maternal effects interact with various postnatal rearing conditions to affect offspring development and phenotype in wild bird population remains elusive. Here we experimentally studied the interactive effects of pre-laying maternal immunization with a bacterial antigen (lipopolysaccharide) and post-hatching rearing conditions, altered by brood size manipulation, on offspring growth and humoral immunity of wild great tits (*Parus major*).

**Results:**

We found that maternal immunization and brood size manipulation interactively affected the growth and specific humoral immune response of avian offspring. Among nestlings reared in enlarged broods, only those that originated from immunized mothers grew better and were heavier at fledging stage compared to those that originated from non-immunized mothers. In contrast, no such effects were observed among nestlings reared in non-manipulated (control) broods. Moreover, offspring of immunized females had a stronger humoral immune response to lipopolysaccharide during postnatal development than offspring of non-immunized females, but only when the nestling was reared in control broods.

**Conclusions:**

This study demonstrates that offspring development and their ability to cope with pathogens after hatching are driven by mutual influences of pathogen-induced prenatal maternal effects and post-hatching rearing conditions. Our findings suggest that immune-mediated maternal effects may have context-dependent influences on offspring growth and immune function, related to the postnatal environmental conditions experienced by the progeny.

**Electronic supplementary material:**

The online version of this article (10.1186/s12983-018-0272-y) contains supplementary material, which is available to authorized users.

## Background

Early life environmental conditions play a key role in determining an individual’s phenotype, with consequences for fitness [1, 2]. Mothers, to a large extent, provide the primary environment experienced by their offspring both before and after birth/hatching. Indeed, females have the potential to create and adjust the prenatal environment and thus affect developmental trajectories of their progeny [3]. Such prenatal maternal effects (MatEs) not only influence embryo growth and development (e.g. [4]), but most importantly, determine morphology, physiology and behaviour of offspring during their postnatal life (e.g. [3, 5, 6]). However, the prenatal environment provided by a mother is, at least to some extent, under the influence of the environmental conditions experienced by her before and during breeding. As a result, mothers can transfer some information about the local environment they experience to the next generation (e.g. [7, 8]). Such environmentally-induced MatEs have been suggested to be a form of adaptation to a heterogeneous but predictable environment, by which females can prepare their progeny for postnatal conditions, to enhance fitness [3, 9]. However, the fitness benefits of prenatal MatEs are primarily expected when the female and her offspring experience the same environmental conditions [2, 9].

Prenatal transfer of antibodies (Abs) from a mother to her progeny is a good example of a MatE by which a female may shape offspring resistance to pathogens and parasites [10–12]. Maternal antibodies (MatAbs) are a primary form of protection against pathogens for neonates, since the lack of a mature and efficient immune system makes them especially prone to infections [13, 14]. Therefore, Ab-mediated MatEs may benefit offspring by helping them to cope with pathogens and parasites and ultimately increase their survival prospects (e.g. [10, 15–17]). However, females can only provide this protection to offspring against pathogenic antigens to which they have been previously exposed (e.g. [18]). Moreover, there is evidence that maternally-derived Abs affect the development of a neonate’s immune system, which has consequences for immune function in both the short- and long-term (e.g. [12]). On the one hand, MatAbs may prime an offspring’s own immunity and thereby induce a stronger humoral immune response to pathogenic antigens encountered by a mother and her progeny [19–21]. On the other hand, maternally-derived Abs have also been observed to both suppress humoral immune response in progeny (e.g. [22–24]) and to be neutral to the function of offspring humoral immunity [25, 26]. MatAbs have also been shown to positively affect postnatal offspring growth (e.g. [12, 27, 28]), especially by allowing offspring to decrease the intensity of costly immune responses (both innate and acquired) to pathogen exposure. Consequently, the amount of resources allocated to the immune system may be reduced and reallocated to growth [10]. Grindstaff’s study [29] validated this hypothesis and showed that MatAbs mitigate the negative effects of offspring antigen exposure on growth during post-hatching development, but only if a mother and her progeny share the same local disease environment.

In fact, the consequences of Ab-mediated MatEs for offspring immunity and/or growth have been shown to be strongly dependent on the extent to which the maternal and offspring disease environments match (e.g. [19, 22, 23, 29]). However, the potential effects of maternally-derived Abs on offspring performance may also be modified by other circumstances. Among these, the postnatal rearing conditions that determine food availability for the offspring seem to be the most important (e.g. [30–34]). Indeed, poor nutritional conditions during postnatal development commonly have a negative effect on offspring growth rate and immune function (e.g. [31, 32, 35]) and result in a physiological trade-off between these two life history traits (e.g. [36–38]). To our knowledge, the effects of maternal immunity transfer on offspring growth and immunity in the context of altered postnatal rearing conditions have been examined to date by Lozano and Ydenberg [28] on wild tree swallows (*Tachycineta bicolour*) and by Ismail et al. [34] on captive feral pigeons (*Columbia livia*). The first study demonstrated that maternal immunization led to faster nestling growth regardless of whether nestlings were reared in enlarged or reduced broods [28]. In the latter study, the authors observed that offspring with lower levels of MatAbs grew better than those with higher MatAbs levels when reared under good food conditions, with no difference when food was restricted [34]. These findings are inconsistent and imply that more studies are needed to understand how maternal immunity transfer affects offspring development under variable postnatal rearing conditions.

The aim of the current study was to determine how pre-laying maternal exposure to a bacterial antigen affects offspring growth and humoral immunity under experimentally altered postnatal rearing conditions in the wild great tit (*Parus major*). We immunized some females with lipopolysaccharide (LPS) prior to egg laying to simulate a natural pathogen infection and elicit an increased LPS-specific Ab transfer to the eggs (e.g. [20, 29]), while other females were not immunized. After hatching, we partially cross-fostered nestlings between pairs of broods that belonged to immunized and non-immunized females. Simultaneously, we manipulated brood size by creating enlarged and non-manipulated (control) broods of immunized and non-immunized females to alter post-hatching rearing conditions. We measured nestling growth, fledgling body size and survival. On day 5 after hatching, we activated the offspring’s immune system by injecting each nestling with LPS across all broods.

We hypothesized that offspring performance would be affected by an interaction between the LPS-induced prenatal MatE and postnatal developmental environment (manipulated brood size) experienced by the progeny. Specifically, we expected that offspring of LPS-immunized mothers would grow better than offspring of non-immunized females, and that the difference would be especially pronounced under harsh rearing conditions. We expected this result for two reasons. First, after offspring exposure to LPS, only the nestlings from LPS-immunized mothers should cope better with this antigen and consequently invest more in growth than in the immune response due to protective effects of LPS-specific MatAbs (see [29]). Second, since an immune response is costly and there is a trade-off between offspring growth and immune function (e.g. [36]), the effect of maternal immunization should be observed under poor rather than control post-hatching conditions. We also expected that the specific immune response to LPS and total antibody production in offspring would generally be larger among nestlings of LPS-immunized females compared to nestlings of non-immunized females, due to a priming of offspring humoral immunity by maternally-derived Abs [10, 20]. However, nestlings of LPS-immunized and non-immunized females reared in enlarged broods should produce less antibodies than those reared in non-manipulated broods due to the resource-draining costs of mounting an immune response.

## Methods

### Study species and site

The study was conducted in 2013–2014 in a nest box-breeding population of great tits located in the Grobelczyk Woodland, a northern part of the Niepołomice Forest, southern Poland (50°06′N 20°24′E). The great tit is a small, sexually dimorphic, hole-nesting passerine bird. In the studied population, female great tits facultatively produce two clutches during each breeding season; mean clutch size ± standard error (SE) in the first and second broods: 10.8 ± 1.2 and 7.7 ± 1.1 eggs, respectively. Only females incubate their eggs for about 13 days. After hatching, nestlings are fed by both parents and fledge within the next 15–18 days [39]. The study site was situated in deciduous woodland predominated by oaks, hornbeams and limes, and included 255 wood nest boxes in 2013 and 233 in 2014 (interior dimensions: 9.0 × 9.0 × 27.5 cm) approximately distributed in a 50 × 40 m grid.

### Procedures and experimental protocol

**Fig. 1:**
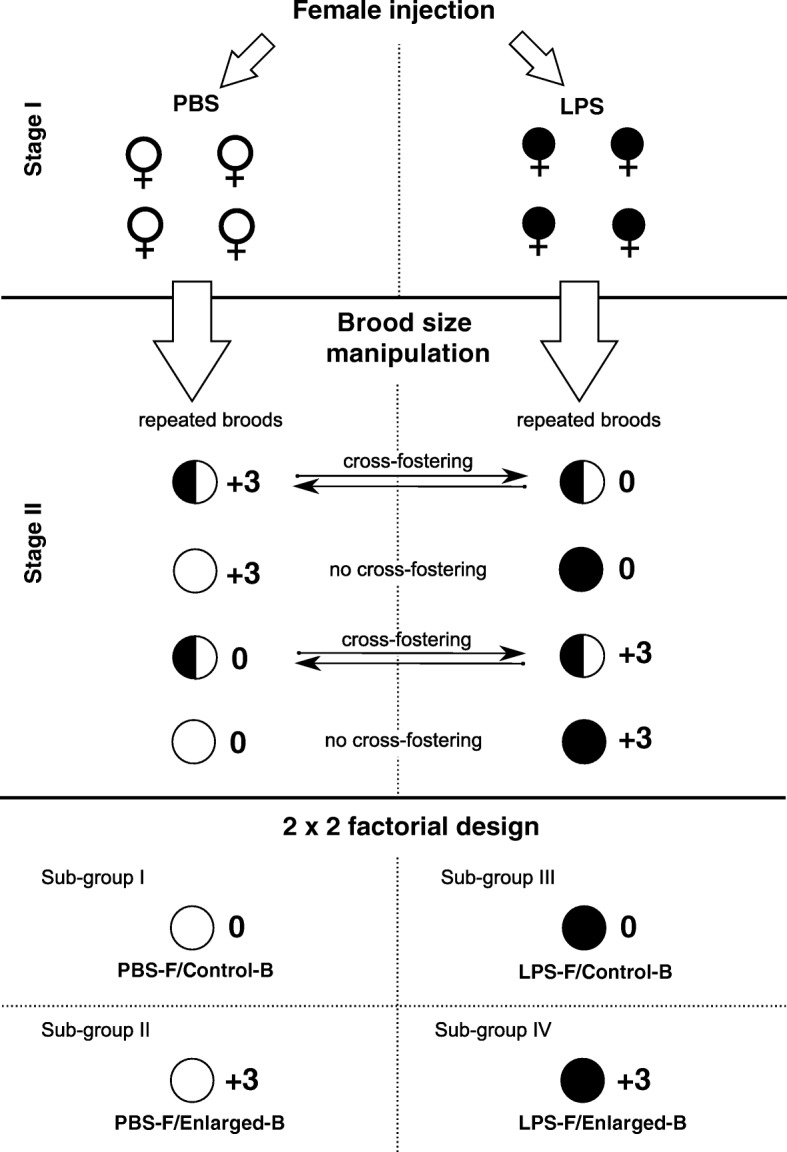
The scheme of the experimental design. Stage I included injection of females with saline (PBS) or lippolysaccharide (LPS) on the day of completion of their first clutches (first clutches with nests were subsequently removed). Stage II included cross-fostering of nestlings between repeated broods of control and LPS-immunized females (a part of the broods could not be cross-fostered, but they were included to dataset), with simultaneous brood size manipulation (half of the broods were enlarged by adding three nestlings, and the other half remained unchanged). As a result, we had a 2 × 2 factorial design for our experiment, with four sub-groups of nestlings: PBS-F/Control-B – nestlings of control females reared in control (non-manipulated) broods, PBS-F/Enlarged-B – nestlings of control females reared in enlarged broods, LPS-F/Control-B – nestlings of LPS-immunized females reared in control broods and LPS-F/Enlarged-B – nestlings of LPS-immunized females reared in enlarged broods

In 2013 and 2014, from the beginning of April, when great tits initiated their first nests in the study area, we regularly monitored nest boxes to detect the exact date the first egg was laid and later to determine clutch size. When females completed their first clutches, we captured them 3.2 ± 0.9 (mean ± SE) days after starting incubation and randomly assigned them either to an experimental or control group. Experimental females (*N* = 50) were inter-abdominally injected with 50 μl of LPS (from *Salmonella enterica* serotype typhimurium; Sigma, Cat. No. L-7261) suspended in phosphate-buffered saline (PBS) using a concentration of 0.1 mg kg body mass^− 1^. Control females (*N* = 54) received 50 μl of PBS via the same procedure (Fig. 1). LPS is a thymus-independent antigen obtained from the outer coat of a gram-negative bacteria that is commonly used to activate bird immune systems in ecological and behavioural research (e.g. [20, 40, 41]) since it stimulates both the innate and acquired immune responses [42], and maternal LPS-specific antibodies are passed onto the eggs and nestlings [24, 29, 43]. The LPS dose in our study was similar or lower compared to doses used in previous bird studies (e.g. [20, 24, 29]). On the day of capture, we measured female body mass to the nearest 0.01 g using an electronic balance, and female tarsus length to the nearest 0.1 mm using an electronic calliper. Each female was individually marked by using numbered aluminium and colourful (alphanumeric) rings. We also drew from each female ca. 75 μl of blood from the brachial vein to determine LPS-specific and total Ab levels. LPS-immunized and control females on the day of capture did not differ in body mass, clutch size, LPS-specific and total Ab levels (for details, see Additional file 1: Table S1). After injection, the existing nest with all eggs was removed from the nest box to ensure that the female would be delayed in her breeding long enough to mount a specific Ab response to LPS immunization [20].

Next, the replacement nest for each female was located and monitored to determine the onset of egg laying and clutch size. Re-nested females were individually recognized during incubation based on alphanumeric rings. Overall, 38 immunized and 42 control females were found to repeat their breeding attempts in the nest boxes; however, three females from the experimental group and one from the control group abandoned their nests during egg laying or incubation. Clutches of re-nested females were visited daily from the day proceeding the expected hatching date to record the actual hatching date and number of hatchlings. To control for the potential effects of maternal immunization on parental provisioning, and to separate prenatal MatEs from postnatal rearing conditions within a brood, we performed a partial cross-fostering of the nestlings on day 2 after hatching (hatching day = 0) between pairs of nests of immunized and control females (Fig. 1). The dyads included only nests with the same hatching date and a similar clutch size (± 1 egg). Just before cross-fostering, we weighed and ranked siblings in relation to their body mass. Afterwards we swapped half of the nestlings according to this mass based-rank (every second nestling was exchanged) to ensure that the cross-fostered offspring reflected the whole range of mass hierarchy observed within the original broods that were assigned to a dyad (see [44]). We were only able to perform cross-fostering for part of the replacement broods (in total 38 nests = 19 dyads) due to the lack of compatibility in hatching date and clutch size between nests. There were no differences in the mean nestling body mass and brood sex ratio 2 days after hatching within a nest before and after cross-fostering (for details, see Additional file 1: Table S2). Since there were a number of broods that could not be matched in dyads, we included a part of such non-cross-fostered nests of LPS-immunized and control females in the dataset (23 total nests) in order to increase sample size and control for the effect of the cross-fostering procedure on offspring performance (details in statistical analysis sub-section; see also [45]; Fig. 1). The other portion of the non-cross-fostered broods were used as donor nests (15 broods; details presented below). The donor nests were chosen randomly from all non-cross-fostered broods and were not included for further analyses.

Concurrently with cross-fostering, we conducted brood size manipulation to create standard differences in rearing conditions among nests of immunized and control females. One randomly selected brood in each dyad was enlarged by adding three nestlings that originated from a donor nest, whereas the other brood within a dyad was not manipulated. Among non-cross-fostered broods, we also enlarged a randomly chosen half of the nests of immunized and control females; the second half remained non-manipulated (Fig. 1). Brood size manipulation to alter rearing conditions within a brood is commonly applied in studies of wild bird populations (e.g. [30, 46, 47]). It has been well-documented that brood enlargement constitutes harsh conditions for offspring and negatively affects their growth and immunity (e.g. [28, 32, 38]) and our findings confirmed this evidence (see Results section for details). The extra nestlings from donor nests were only used to increase within-nest competition and were not included in analyses. Consequently, after performing all the procedures, there were four sub-groups of nestlings that resulted from our experimental setting (Fig. 1).

To recognize nestlings individually, we marked them by clipping nails 2 days after hatching, and we subsequently repeated this procedure on 5-day-old nestlings by using the same code as on day 2. This action enabled individual identification of each nestling on day 14 after hatching (at which point we marked nestlings using numbered aluminium rings). We measured nestling body mass on day 2, 5 and 14 after hatching to the nearest 0.01 g using an electronic balance. Thus, we were able to assess offspring growth at an early and late stage of its development (such a division was helpful for interpreting results before and after offspring immunization; see [29] and details below). Early growth rate was calculated as (body mass on day 5 - body mass on day 2)/3 and late growth rate as (body mass on day 14 - body mass on day 5)/9, with both values expressed as the gain of body mass per day (see [44]). On day 14 after hatching, we also measured nestling tarsus length to the nearest 0.1 mm using an electronic calliper. Moreover, 5 days after hatching, all nestlings (in all sub-groups) were inter-abdominally injected with 25 μl of LPS suspended in PBS at a concentration of 0.1 mg kg body mass^− 1^ [20]. Immediately prior to immunization on day 5, and again on day 14 after hatching (9 days after immunization), we drew from each nestling ca. 50 μl of blood from the brachial vein to assess LPS-specific and total Ab levels. The time between offspring immunization and repeated blood sampling was chosen so that offspring could produce Abs against LPS [20, 48]. Both nestlings from cross-fostered broods (either moved to foster nests or kept in original nests) and nestlings from non-cross-fostered broods (remained in original nests) were handled in the same way, so that offspring who originated from each sub-group were treated as similarly as possible.

### Humoral immunity assessment

We determined humoral immunity of females and their offspring by quantifying LPS-specific and total Ab levels. Briefly, blood taken from females or nestlings was put into heparinized capillaries and kept cold until its return to the laboratory. Capillaries with blood were centrifuged at 1300 rpm for 7 min to separate plasma from blood cells. Plasma samples were stored at − 20 °C until further analysis of LPS-specific and total Ab levels, which we performed using an enzyme-linked immunosorbent assay (ELISA, e.g. [29]; for details of analyses, see Additional file 2). All plasma samples were quantified within 2–3 months after sample collection, and each season was quantified separately (this fact was controlled in statistical analyses as the year effect).

### Molecular sexing

We used a cellular fraction of blood (after centrifugation) that was gathered on day 5 after hatching to obtain a DNA sample from nestlings. We also used DNA samples obtained from tissue taken from nestlings that died before day 5 and dead embryos from unhatched eggs. Blood and tissue samples were kept in 0.5 ml of 96% ethanol and stored at room temperature. DNA was extracted using the Chelex method, and then two homologous genes (*CHD1-W* and *CHD1-Z*) were amplified following the protocol of Griffiths et al. [49]. Products were separated by electrophoresis on 3% agarose gels, which were stained with Redgell and visualized under UV transillumination. Nestling sex was assessed according to the presence of one band for males or two bands for females.

### Statistical analysis

First, we analyzed the effect of maternal immunization on the primary reproductive effort of females. We used general linear models to compare differences in the number of days needed to start egg laying in replacement nests, hatching success and the primary offspring sex ratio between control and LPS-immunized females. We calculated hatching success as the proportion of eggs hatched relative to clutch size and the offspring sex ratio as the proportion of males relative to brood size. We also used a repeated-measures analysis of variance to determine changes in pre- and post-immunization clutch sizes between control and LPS-immunized females. All models contained two fixed factors: group (control vs. immunized females) and year to control for inter-season effects.

Thereafter we examined how pre-laying maternal immunization, post-hatching brood size manipulation and interaction between these two factors affected offspring performance. Specifically, we fitted several linear mixed models to analyze the effects of the treatments on hatchling body mass, early and late nestling growth, fledgling body mass and tarsus length, LPS-specific and total Ab level in 5-day-old nestlings, LPS-immune response and total Ab production following offspring immunization (the latter two values were estimated as the difference between post- and pre-immunization Ab titres). Full models included year (to control for inter-season effects), maternal immunization, brood size manipulation, offspring sex (to account for sex-specific variation in offspring traits) and nestling status as fixed factors. Nestling status controlled for potential consequences of the cross-fostering procedure on nestling traits. For example, some differences in nest environment that result from ectoparasite presence (e.g. [8]) and specific microbiomes (e.g. [50]), and methodological biases caused by non-random breeding and changes to brood composition [45], can affect offspring performance independently of experimental treatments. Following the suggestions of Winney et al. [45], for better statistical control of potential biases produced by cross-fostering we decided to distinguish three types of nestlings in our experiment (three levels of nestling status factor): nestlings from cross-fostered nests that were moved to foster broods, nestlings from cross-fostered nests that remained in their original broods and nestlings from non-cross-fostered nests where no changes in brood composition were made (all nestlings stayed in their original nests). In addition, each initial model contained clutch size, hatching date, hatchling body mass (except for the analysis that examined nestling body mass 2 days after hatching), LPS-specific and Ab level on day 5 after hatching (only in the analyses that examined LPS-immune response and total Ab production, respectively) as covariates to control for their potential effects on offspring performance.

To analyze the effects of treatments on offspring survival, from hatching to day 14 we fitted a generalized linear model with a logit-link function and binomial error variance. The full model included the same explanatory variables like those models that analyzed morphological or physiological nestling traits.

In all mixed models we tested a two-way interaction between maternal immunization and brood size manipulation, the primary term of interest in our study. However, to determine whether the sexes responded differently to prenatal and postnatal conditions following treatments, we also tested two-way interactions between offspring sex and maternal immunization and brood size manipulation. To reduce the full models, we sequentially eliminated non-significant interactions and covariates (if *P* ≥ 0.10), beginning with the least significant terms. If there was a significant interaction term, we performed post hoc pair-wise comparisons of the marginal means to separate the simple main effects involved in the interaction (by comparing the level of one factor within levels of another factor; [51, 52]). Moreover, to control for non-independence of offspring that originated from different original broods but were reared in the same foster broods, we included nest of origin (female identity) and nest of rearing (foster female identity) in all models as random factors. All mixed model analyses were based on restricted maximum likelihood (REML) estimations, and denominator degrees of freedom were approximated by the Satterthwaite method.

We checked for normality and homoscedasticity of residuals derived from the models using normal distribution error variance. Finally, to meet assumptions, we had to transform the following variables: late nestling growth (square-transformed), LPS-specific Ab level in 5-day-old nestlings (coded by adding 1 and log-transformed), total Ab level in 5-day-old nestlings, LPS-immune response and total Ab production (all log-transformed). All statistical tests were two-tailed with the significance level set at *P* ≤ 0.05 and were performed in SPSS version 24.0 (IBM Corp.). There were different sample sizes between analyses because of abandoned broods by females, predation on females and nestlings, or missing collected blood samples. The marginal mean ± SE for categorical factors and parameter estimate ± SE for covariates are presented throughout the text. In the case of late nestling growth, data are presented as re-transformed mean ± SE.

## Results

### Primary reproductive effort of females

Maternal immunization had no effect on the time needed to re-initiate egg laying after treatment (control vs. immunized: 5.9 ± 0.2 and 6.2 ± 0.2 days; group: F_1, 58_ = 0.79, *P* = 0.377; year: F_1, 58_ = 0.08, *P* = 0.786). Re-nested control and LPS-immunized females did not differ in their clutch sizes (control vs. immunized: 10.4 ± 0.2 and 10.6 ± 0.2 eggs; group: F_1, 116_ = 0.08, P = 0.78; year: F_1, 116_ = 3.48, *P* = 0.067), although there were season-dependent differences in clutch size between the first and replacement broods (clutch order × year: F_1, 116_ = 69.60, *P* <  0.001). Additionally, neither hatching success (the proportion of hatched eggs for control vs. immunized: 0.84 ± 0.02 and 0.87 ± 0.02; group: F_1, 58_ = 0.87, *P* = 0.354; year: F_1, 58_ = 1.95, *P* = 0.168) nor primary offspring sex ratio (the proportion of males for control vs. immunized: 0.55 ± 0.06 and 0.59 ± 0.06; group: F_1, 53_ = 0.23, *P* = 0.634; year: F_1, 53_ = 1.89, *P* = 0.175) differed between control and LPS-immunized females.

### Hatchling body mass and nestling growth

Maternal immunization did not affect nestling body mass 2 days after hatching. This finding indicated that initial nestling body mass was similar regardless of whether they originated from control or LPS-immunized mothers (control vs. immunized: 2.73 ± 0.08 and 2.93 ± 0.08 g; F_1, 62.18_ = 2.91, *P* = 0.093; *N* = 375). We also found no differences in initial body mass between nestlings assigned to control and enlarged broods (control vs. enlarged: 2.79 ± 0.07 and 2.87 ± 0.07 g; F_1, 288.38_ = 0.63, *P* = 0.429; F_1, 288.38_ = 0.63, *P* = 0.429; *N* = 375). More importantly, we did not find any interactive effect of maternal immunization and brood size manipulation on initial hatchling body mass (F_1, 285.80_ = 0.17, *P* = 0.678; *N* = 375), which meant that nestling body mass 2 days after hatching was similar among the four experimental sub-groups. The effects of year, offspring sex and nestling status on hatchling body mass were also not significant (all *P* ≥ 0.084).

Offspring growth rate measured before LPS immunization (i.e. early nestling growth) was higher among nestlings hatched in 2013 than those hatched in 2014 (1.33 ± 0.04 and 1.06 ± 0.05 g per day, respectively; Table 1). Male offspring grew faster compared to female offspring regardless of maternal immunization and brood size manipulation (1.21 ± 0.01 and 1.19 ± 0.01 g per day, respectively; Table 1). Moreover, early nestling growth was negatively correlated to hatching date (− 0.03 ± 0.01, Table 1) and positively to hatchling body mass (0.26 ± 0.01, Table 1).

**Table 1:** Results of analyses on early and late nestling growth, fledgling body mass and tarsus length

Sources of variation	df	F	P
Early nestling growth (g per day); *N* = 352			
Year	1, 51.28	9.74	0.003
Maternal immunization	1, 21.43	0.09	0.767
Brood size manipulation	1, 46.48	0.93	0.339
Nestling status	2, 83.99	0.12	0.888
Offspring sex	1, 300.93	6.80	0.010
Hatching date	1, 50.68	5.33	0.025
Hatchling body mass	1, 302.87	488.34	< 0.001
Square-transformed late nestling growth (g per day); *N* = 281			
Year	1, 37.99	0.06	0.805
Maternal immunization	1, 22.13	0.80	0.381
Brood size manipulation	1, 34.93	11.96	0.001
Nestling status	2, 73.25	0.75	0.474
Offspring sex	1, 244.12	28.12	< 0.001
Hatchling body mass	1, 247.91	339.78	< 0.001
Maternal immunization × brood size manipulation	1, 230.37	7.47	0.007
Fledgling body mass (g); *N* = 281			
Year	1, 36.13	3.84	0.058
Maternal immunization	1, 20.71	0.55	0.468
Brood size manipulation	1, 39.20	18.96	< 0.001
Nestling status	2, 66.04	0.54	0.586
Offspring sex	1, 233.14	50.85	< 0.001
Hatchling body mass	1, 242.28	26.94	< 0.001
Maternal immunization × brood size manipulation	1, 226.81	7.78	0.006
Maternal immunization × offspring sex	1, 230.44	2.99	0.085
Fledgling tarsus length (mm); *N* = 281			
Year	1, 30.46	0.04	0.842
Maternal immunization	1, 35.73	2.86	0.099
Brood size manipulation	1, 32.31	1.84	0.184
Nestling status	2, 78.58	0.64	0.530
Offspring sex	1, 259.34	33.00	< 0.001
Hatchling body mass	1, 263.82	6.21	0.013
Maternal immunization × brood size manipulation	1, 232.86	5.83	0.017

Table 1 presents the results of linear mixed models that examined the effects of a set of explanatory variables on early nestling growth, square-transformed late nestling growth, fledgling body mass and fledgling tarsus length. Full models included year (to control for inter-season differences), maternal immunization (control vs. immunized females), brood size manipulation (control vs. enlarged broods), nestling status (to control for cross-fostering effects; there were three levels of this factor: nestlings from cross-fostered nests moved to foster broods, nestlings from cross-fostered nests that stayed in their original broods and nestlings from non-cross-fostered nests where all nestlings stayed in their original nests) and offspring sex (to control for differences between male and female nestlings) as fixed factors, and hatching date, clutch size and hatchling body mass (2 days after hatching) as covariates. All two-way interaction terms between maternal immunization, brood size manipulation and offspring sex were tested as well. Nest of origin (female identity) and nest of rearing (foster female identity) were included in all models as random factors (results not shown). Presented are reduced models after sequential backward elimination of non-significant (if P ≥ 0.10) interactions and covariates

**Fig. 2:**
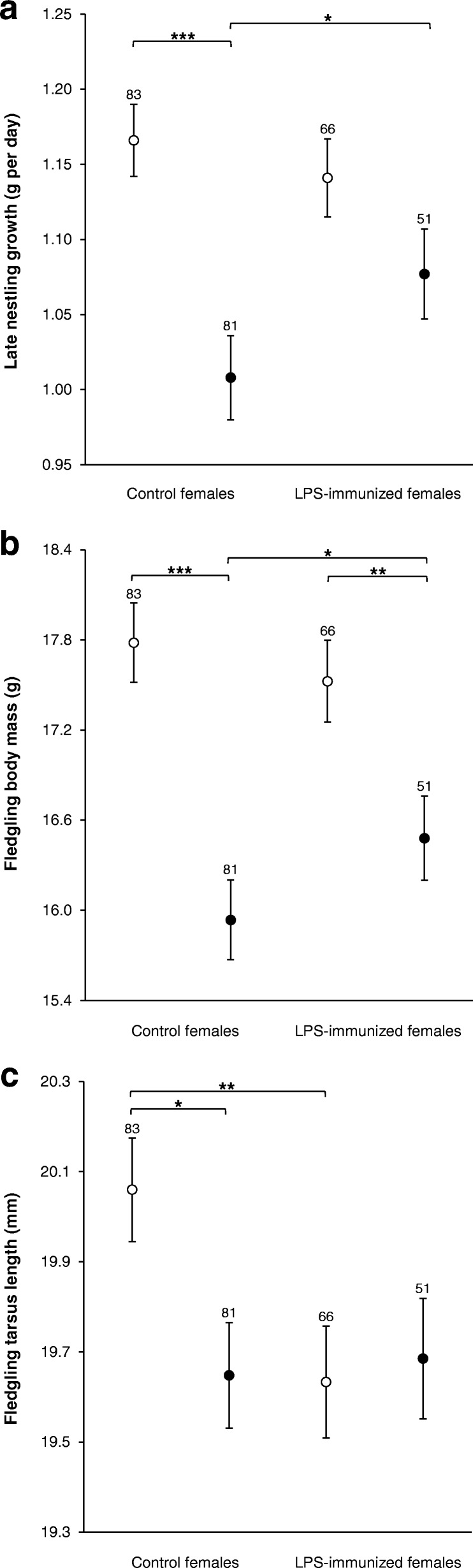
Re-transformed late nestling growth (**a**), fledgling body mass (**b**) and tarsus length (**c**) in relation to maternal immunization (control vs. LPS-immunized females) and brood size manipulation (control vs. enlarged broods). Least square mean ± SE derived from final models are shown. Open circles denote control broods, whereas filled circles denote enlarged broods. Sample sizes are noted above bars. Significant pair-wise differences are marked by * (*P* <  0.05), ** (*P* <  0.01) or *** (*P* <  0.001)

Offspring growth measured after LPS immunization (i.e. late nestling growth) was affected by interactive effects of maternal immunization and brood size manipulation (Table 1). This interaction meant that offspring of LPS-immunized females grew faster than offspring of control females when raised in enlarged broods, while no such effect was observed in control broods (Table 3, Fig. 2a). Furthermore, the offspring of control females reared in enlarged broods grew slower than those reared in control broods (Table 3, Fig. 2a). Offspring of LPS-immunized mothers reared in enlarged broods also tended to grow slower than those reared in control broods (Table 3, Fig. 2a). Late nestling growth was faster in male compared to female offspring regardless of treatments (1.14 ± 0.02 vs. 1.06 ± 0.02 g per day, respectively; Table 1) and was negatively correlated with hatchling body mass (− 0.35 ± 0.02, Table 1).

### Fledgling body mass and size

Fledgling body mass was explained by an interaction between maternal immunization and brood size manipulation (Table 1). This interaction exhibited a similar pattern to that observed for late nestling growth (Fig. 2a, b). The offspring of LPS-immunized mothers were heavier than offspring of control females when reared in enlarged broods but not when reared in control broods (Table 3, Fig. 2b). Moreover, offspring of LPS-immunized females reared in enlarged broods had lower fledgling body mass compared to those reared in control broods (Table 3, Fig. 2b). Such differences were also observed between offspring of control mothers reared in enlarged or control broods (Table 3, Fig. 2b). Male and female fledglings differed in body mass (17.35 ± 0.19 and 16.51 ± 0.19 g, respectively; Table 1), and fledgling body mass was positively correlated with body mass at hatching (0.39 ± 0.07, Table 1).

Fledgling tarsus length was also affected by interactive effects of maternal immunization and brood size manipulation (Table 1). This effect resulted from the fact that offspring of LPS-immunized females had shorter tarsi than offspring of control females when reared in control broods but not when reared in enlarged broods (Table 3, Fig. 2c). Moreover, the offspring of control mothers reared in control broods had longer tarsi compared to those reared in enlarged broods, with no such effects among the offspring of LPS-immunized females (Table 3, Fig. 2c). Male fledglings had larger tarsi than female ones regardless of treatments (20.0 ± 0.1 vs. 19.5 ± 0.1 mm, respectively; Table 1). Fledgling tarsus length was positively correlated with hatchling body mass (0.13 ± 0.05, Table 1).

### LPS-specific immune response and total Ab production in offspring

**Fig. 3:**
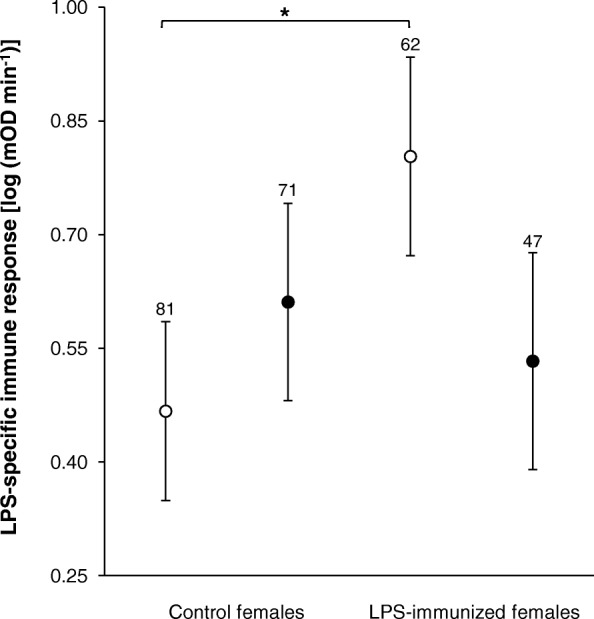
Log-transformed LPS-specific immune response in offspring (estimated as the difference between post- and pre-immunization Ab titres and expressed as mOD min^− 1^) in relation to maternal immunization (control vs. LPS-immunized females) and brood size manipulation (control vs. enlarged broods). Least square mean ± SE derived from the final model are shown. Open circles denote control broods, whereas filled circles denote enlarged broods. Sample sizes are noted above bars. Significant pair-wise difference is marked by * (*P* <  0.05)

Most nestlings (83%) had detectable LPS-specific Abs on day 5 after hatching. Maternal immunization, brood size manipulation, offspring sex, year and nestling status did not affect LPS-specific Ab levels in 5-day-old nestlings (all *P* ≥ 0.260). However, LPS-specific Ab level was positively correlated with hatchling body mass (0.13 ± 0.02; F_1, 250.24_ = 63.13, *P* <  0.001, *N* = 268). Almost all offspring (98%) responded to LPS immunization by increasing LPS-specific Ab production from day 5 to day 14 after hatching. Offspring LPS immune response was affected by maternal immunization and brood size manipulation interaction (Table 2, Fig. 3). Follow-up tests revealed that offspring of LPS-immunized females had higher specific immune responses to LPS than offspring of control females, but only when reared in control broods (Table 3, Fig. 3). Moreover, the LPS-immune response among offspring of LPS-immunized females tended to be stronger in nestlings reared in control compared to enlarged broods (Table 3, Fig. 3). Further, there were no differences in the LPS-immune response between offspring of control females reared in control and enlarged broods or offspring of control and LPS-immunized females reared in enlarged broods (Table 3, Fig. 3). The specific immune response of offspring was stronger in 2014 than in 2013 (1.37 ± 0.11 and − 0.17 ± 0.12 [log (mOD min^− 1^)], respectively) and depended on nestling status (Table 2). Post hoc multiple comparisons with *P*-value correction showed that the LPS-immune response among swapped nestlings tended to be lower compared to nestlings that stayed in their original cross-fostered broods (0.42 ± 0.11 and 0.67 ± 0.11 [log (mOD min^− 1^)], respectively; F_1, 249.10_ = 3.38, *P* = 0.067). There were no differences between swapped nestlings and nestlings from non-cross-fostered broods (0.42 ± 0.11 vs. 0.73 ± 0.14 [log (mOD min^− 1^)], respectively; F_1, 44.96_ = 1.36, *P* = 0.250), or between nestlings that stayed in their original cross-fostered broods and those from non-cross-fostered broods (0.66 ± 0.11 and 0.73 ± 0.14 [log (mOD min^− 1^)], respectively; F_1, 43.96_ = 0.00, *P* = 0.964).

**Table 2:** Results of analyses on LPS-specific immune response and total Ab production in offspring

Sources of variation	df	F	P
Log-transformed LPS-specific immune response (mOD min^− 1^); *N* = 261			
Year	1, 40.70	91.51	< 0.001
Maternal immunization	1, 50.39	0.72	0.402
Brood size manipulation	1, 8.73	0.37	0.557
Nestling status	2, 82.04	3.26	0.043
Offspring sex	1, 222.74	3.39	0.067
Hatchling body mass	1, 234.87	2.88	0.091
Maternal immunization × brood size manipulation	1, 239.95	4.81	0.029
Maternal immunization × offspring sex	1, 221.43	3.11	0.079
Log-transformed total Ab production (mOD min^−1^); *N* = 257			
Year	1, 42.67	21.40	< 0.001
Maternal immunization	1, 58.05	0.41	0.524
Brood size manipulation	1, 244.24	0.29	0.588
Nestling status	2, 82.66	0.04	0.961
Offspring sex	1, 228.09	1.22	0.270
Hatchling body mass	1, 238.20	3.63	0.058

Table 2 presents the results of linear mixed models that examined the effects of a set explanatory variables on log-transformed LPS-specific immune response and log-transformed total Ab production (both specific immune response and total Ab production were estimated as the differences between post- and pre-immunization Ab titres). Full models included year (to control for inter-season differences), maternal immunization (control vs. immunized females), brood size manipulation (control vs. enlarged broods), nestling status (to control for cross-fostering effects; there were three levels of the factor: nestlings from cross-fostered nests moved to foster broods, nestlings from cross-fostered nests that stayed in their original broods and nestlings from non-cross-fostered nests where all nestlings stayed in their original nests) and offspring sex (to control for differences between male and female nestlings) as fixed factors, and hatching date, clutch size, hatchling body mass (2 days after hatching), log-transformed LPS-specific Ab titres (only in the analysis of LPS-specific immune response) and log-transformed total Ab titres (only in the analysis of total antibody production) in 5-day-old nestlings as covariates. All two-way interaction terms between maternal immunization, brood size manipulation and offspring sex were tested as well. Nest of origin (female identity) and nest of rearing (foster female identity) were included in all models as random factors (results not shown). Presented are reduced models after sequential backward elimination of non-significant (if *P* ≥ 0.10) interactions and covariates

**Table 3:** Results of post hoc pair-wise comparisons of significant interactions from linear mixed models

Pair-wise comparisons	df	F	P
Square-transformed late nestling growth (g per day)			
PBS-F/Control-B vs. PBS-F/Enlarged-B	1, 47.94	19.46	< 0.001
PBS-F/Control-B vs. LPS-F/Control-B	1, 49.83	0.95	0.334
PBS-F/Enlarged-B vs. LPS-F/Enlarged-B	1, 39.35	5.18	0.028
LPS-F/Control-B vs. LPS-F/Enlarged-B	1, 59.76	3.17	0.080
Fledgling body mass (g)			
PBS-F/Control-B vs. PBS-F/Enlarged-B	1, 48.14	26.54	< 0.001
PBS-F/Control-B vs. LPS-F/Control-B	1, 46.43	1.22	0.275
PBS-F/Enlarged-B vs. LPS-F/Enlarged-B	1, 36.06	4.72	0.037
LPS-F/Control-B vs. LPS-F/Enlarged-B	1, 58.97	8.19	0.006
Fledgling tarsus length (mm)			
PBS-F/Control-B vs. PBS-F/Enlarged-B	1, 51.29	6.91	0.011
PBS-F/Control-B vs. LPS-F/Control-B	1, 75.86	8.76	0.004
PBS-F/Enlarged-B vs. LPS-F/Enlarged-B	1, 67.36	0.06	0.809
LPS-F/Control-B vs. LPS-F/Enlarged-B	1, 71.89	0.09	0.761
Log-transformed LPS-specific immune response (mOD min^−1^)			
PBS-F/Control-B vs. PBS-F/Enlarged-B	1, 31.52	1.06	0.310
PBS-F/Control-B vs. LPS-F/Control-B	1, 74.48	4.06	0.047
PBS-F/Enlarged-B vs. LPS-F/Enlarged-B	1, 79.69	0.17	0.680
LPS-F/Control-B vs. LPS-F/Enlarged-B	1, 23.13	3.73	0.066
Log-transformed total Ab level in nestlings on day 5 (mOD min^−1^)			
Female-N/Control-B vs. Female-N/Enlarged-B	1, 26.56	0.89	0.353
Female-N/Control-B vs. Male-N/Control-B	1, 235.37	0.11	0.736
Female-N/Enlarged-B vs. Male-N/Enlarged-B	1, 223.01	6.54	0.011
Male-N/Control-B vs. Male-N/Enlarged-B	1, 19.18	1.41	0.249

Table 3 presents results of post hoc pair-wise comparisons performed for all significant interactions produced by linear mixed models that analyzed square-transformed late nestling growth, fledgling body mass and tarsus length, log-transformed LPS-specific immune response and log-transformed total antibody level in 5-day-old nestlings to separate the simple main effects involved in those interactions (by comparing the level of one factor within levels of another factor). Explanations of abbreviations: PBS-F/Control-B – nestlings of control females reared in control (non-manipulated) broods, PBS-F/Enlarged-B – nestlings of control females reared in enlarged broods, LPS-F/Control-B – nestlings of LPS-immunized females reared in control broods, LPS-F/Enlarged-B – nestlings of LPS-immunized females reared in enlarged broods, Female-N/Control-B – female nestlings reared in control broods, Female-N/Enlarged-B – female nestlings reared in enlarged broods and Male-N/Control-B – male nestlings reared in control broods, Male-N/Enlarged-B – male nestlings reared in enlarged broods

Total Ab level in 5-day-old nestlings was explained by an interaction between brood size manipulation and offspring sex (F_1, 229.30_ = 4.44, *P* = 0.036; *N* = 257). This interaction meant that male nestlings had higher levels of total Abs compared to female nestlings, but only when reared in enlarged broods (− 0.85 ± 0.15 and − 1.22 ± 0.16 [log (mOD min^− 1^)], respectively; Table 3) and not when reared in control broods (− 1.08 ± 0.14 and − 1.03 ± 0.14 [log (mOD min^− 1^)], respectively; Table 3.). Only year (total Ab production was higher in 2014 than in 2013; 1.81 ± 0.08 and 1.27 ± 0.09 [log (mOD min^− 1^)], respectively) and hatchling body mass (it tended to be positively correlated with total Ab production; 0.07 ± 0.04) affected offspring total Ab production (Table 2).

### Offspring survival

Maternal immunization, brood size manipulation, offspring sex, year or nestling status did not influence the survival of the offspring (all *P* ≥ 0.355; *N* = 297). Only hatchling body mass tended to be a positive predictor of offspring survival (0.67 ± 0.06; F_1, 289_ = 3.54, *P* = 0.061).

## Discussion

Our study demonstrated that maternal immunization did not affect the initiation of replacement clutches, clutch size, hatchling body mass or offspring sex ratio. Therefore, postnatal offspring development was unlikely to be influenced by changes in female condition, overall investment in eggs and brood composition. Given that we performed cross-fostering between broods of LPS-immunized and control mothers, the influence of maternal exposure to LPS on offspring performance is also not likely to be affected by potential carry-over effects (e.g. maternal provisioning behaviour). Thus, the observed effects of maternal treatment on offspring growth and immunity result primarily from changes in egg quality.

We found no effects of maternal immunization and brood size manipulation on early nestling growth. On one hand, this result may suggest that the period during which we measured growth (from 2 to 5 days after hatching) was not long enough to detect the potential effects of the treatments. On the other hand, we could fail to find some effects of the treatments on early nestling growth as the changes in nestling growth observed between day 5 and 14 after hatching were most probably caused by offspring immunization.

In accordance with our predictions, we showed that late nestling growth was interactively influenced by maternal immunization and post-hatching rearing conditions experienced by the offspring. Nestlings reared in broods with harsh postnatal conditions generally grew slower than those reared in broods with favourable conditions, regardless of maternal exposure to LPS. This finding is consistent with previous studies that documented negative effects of adverse rearing conditions, following brood enlargement, on nestling growth and/or fledging body mass in birds (e.g. [28, 30, 35]). Most importantly, our study revealed a difference in growth between nestlings that originated from LPS-immunized and control mothers, but only when nestlings were reared under a harsh nest environment; offspring of LPS-immunized females exhibited faster growth than the offspring of control females. LPS immunization likely drained nutrients needed for nestling growth due to a costly inflammatory response activation [53, 54], but even then nestlings from LPS-immunized females were able to better cope with that antigenic challenge. Offspring of LPS-immunized mothers were possibly more efficient at eliminating LPS without invoking a strong innate immune response. Indeed, Klasing and Leshchinsky [54] documented that chicks of Japanese quails (*Coturnix japonica*), whose mothers were immunized with LPS, exhibited decreased activity of inflammatory cytokines.

Our results on late nestling growth correspond with previous studies and confirm that immune-mediated MatEs may reduce the negative consequences of early life infections during rapid offspring growth. Briefly, the study on laboratory Japanese quails showed that pre-laying immunization of mothers with LPS or killed avian reovirus (AR) antigen allowed their chicks to mitigate growth-suppressive effects after LPS or AR exposure [29]. In turn, Buechler et al. [27] demonstrated in the wild great tits that nestlings that originated from females exposed to fleas prior to egg laying were heavier at fledging stage, and this effect was mediated by increased MatAb transfer to eggs. Contrary to that research, Lozano and Ydenberg [28] not only immunized females prior to egg laying but also manipulated postnatal rearing conditions (enlarged vs. reduced broods). They found that offspring of females immunized with sheep red blood cells grew faster than those of control females, but brood size manipulation did not interact with maternal treatment. However, recent research by Ismail et al. [34] have shown that although offspring from females immunized with keyhole limpet haemocyanin (KLH) antigen received more anti-KLH Abs than offspring of control females, they did not grow better after KLH exposure when reared under restricted food conditions. In contrast, offspring from control females grew faster, but only when reared in the ad libitum food treatment. Here we showed for the first time that the effects of maternal immunization may be especially beneficial for offspring rearing under harsh post-hatching conditions. In fact, the fitness costs of nestling exposure to pathogens are higher under poor nutritional conditions due to the trade-off between growth and immune function (e.g. [35, 37, 38]); therefore, immune-mediated MatEs may boost offspring reared in poor postnatal environments. Interestingly, there was little difference between nestlings of LPS-immunized and non-immunized females reared in control broods. According to our predictions, nestlings from control females, although reared under a favourable post-hatching environment, should have exhibited reduced growth compared to nestlings from LPS-immunized females. This may result from the fact that offspring of LPS-immunized females responded more strongly to LPS (the higher anti-LPS Ab production) and thus could be limited to invest more resources in growth.

Both fledgling body mass and tarsus length were also affected by the interaction of the treatments. Fledgling body mass had the same pattern as that of late nestling growth. This finding indicates that the interactive effects of maternal immunization and post-hatching conditions on nestling growth persisted until the fledging stage. Fledgling body mass is a fitness-related trait and has been shown to be a strong positive predictor of first-year survival and recruitment in great tit offspring (e.g. [55–58]). Such a result implies that the interaction of prenatal MatEs and postnatal rearing conditions not only has short-term influences on offspring performance but, more importantly, may result in long-lasting fitness consequences. Interestingly, fledgling tarsus length showed a different pattern from that observed for fledgling body mass. Offspring of control females reared in control broods had longer tarsi than the same offspring reared in enlarged broods. This result suggests that harsh rearing conditions after hatching also negatively affected skeletal growth. In fact, harsh postnatal rearing conditions have been previously shown to decrease fledgling tarsus size (e.g. [33, 59]). However, we also found that nestlings from LPS-immunized females reared in control broods had small tarsi similar to nestlings from LPS-immunized and control females reared in enlarged broods. This finding may suggest that maternal immunization affected a strategy of resource allocation in nestlings. In fact, offspring of LPS-immunized females reared in ‘optimal’ conditions grew fast and also had the highest LPS-specific immune response among all groups of nestlings. Thus, smaller tarsus size of fledglings from LPS-immunized mothers may possibly be a consequence of the change in resource partitioning between different demands that can promote body mass gain and immunity development at the expense of skeletal growth.

The specific humoral immune response of offspring to LPS was explained by an interactive effect of maternal immunization and brood size manipulation, despite the fact that the level of LPS-specific Abs in offspring plasma on day 5 was not affected by the treatments. Offspring of LPS-immunized females had stronger immune response than the offspring of control females when reared under ‘optimal’ postnatal conditions. This result indicates that LPS-specific MatAbs might prime the offspring’s own immunity to LPS, which would allow nestlings to mount a stronger response to the antigen after their postnatal immunization (see [20] for details on the mechanisms of this priming). Indeed, such positive effects of MatAbs on the offspring humoral immune response have also been reported previously in other bird species [19–21]. We also observed that among offspring of LPS-immunized females, only those reared in control broods exhibited a higher LPS-specific immune response than those reared in enlarged broods. This result may suggest that the adverse postnatal food conditions experienced by the nestlings had a negative influence on offspring humoral response even though their immunity was primed by MatAbs. This supposition makes sense because under harsh rearing conditions, offspring may invest more in growth than in immune function, especially when they can cope with antigenic immune challenges without the involvement of all immunological defence mechanisms (due to the protective and/or priming effects of MatAbs, e.g. [29]). Maternal immunization and post-hatching rearing conditions did not influence total non-specific Ab production between day 5 and 14 after hatching. However, we found an interactive effect of brood size manipulation and offspring sex on total Ab level on day 5 after hatching. Male and female nestlings can differ in immune function, including total non-specific antibody production (e.g. [60, 61]), and in their developmental responses to post-hatching rearing conditions (e.g. [33, 39]). This fact may explain the interaction between Ab production and postnatal rearing conditions that we observed among 5-day-old offspring.

The observed effects of maternal immunization on offspring growth and humoral immunity found in our study seem to be immune-mediated, but we have limited possibilities to prove it directly. Unfortunately, we did not examine MatAb content in eggs and we also found no differences in MatAb levels among 5-day-old offspring of LPS-immunized and control females. However, a number of studies have shown that maternal immunization with LPS prior to egg laying is an efficient means to increase the transfer of LPS-specific MatAbs to offspring via eggs (e.g. [24, 29, 43]). Therefore, we may expect that nestlings of LPS-immunized females might have had higher initial levels of LPS-specific Abs than nestlings of control females (i.e. only within first days after hatching). After this time, those initial differences disappeared and this is why they were no longer detectable on day 5 (see [20, 26, 62]). It is possible that even such short-term differences could be responsible for the priming of an offspring’s own immunity, and could influence nestling growth and humoral immunity. On the other hand, maternal exposure to LPS may affect female Ab profile and also alter her hormonal state; these changes could result in, for example, enhanced deposition of maternal corticosterone in eggs [63], with further consequences for offspring growth [64]. Therefore, we cannot exclude potential confounding effects of maternal immunization on offspring performance in our study.

## Conclusion

Our study demonstrated that offspring performance is determined by an interaction between the prenatal and postnatal environments. Importantly, we showed that pathogen-induced prenatal MatEs have different consequences for offspring growth and immunity under altered and non-manipulated postnatal rearing conditions. Our findings also confirmed previous evidence that maternal exposure to a pathogen prior to egg laying has the potential to prime an offspring’s own immunity to encounter to the same pathogen, by which the offspring is enabled to better cope with postnatal infections. However, when post-hatching rearing conditions are favourable, such a priming effect may lead to the mounting of a strong Ab immune response in the offspring to specific pathogens. In contrast, when postnatal rearing conditions are poor, the priming effect may only allow for a reduction in the negative effects of early pathogen exposure on offspring growth. Our results suggest that the potential effects of prenatal MatAb transfer on offspring growth and immune function may be context-dependent, i.e. the postnatal environmental conditions experienced by the developing offspring.

## Additional files

Additional file 1: Supplementary materials. Included are 2 supplementary tables (Tables S1 and S2). (DOCX 16 kb)

Additional file 2: Supplementary materials. Included are detailed descriptions of ELISA assays performed to assess LPS-specific and total antibody levels. (DOCX 14 kb)

## Abbreviations

Ab or Abs: Antibody or antibodies
LPS: Lipopolysaccharide
MatAb or MatAbs: Maternal antibody or maternal antibodies
MatE or MatEs: Maternal effect or maternal effects
SE: Standard error
