# Supplementary material for: The effect of pre-laying maternal immunization on offspring growth and immunity differs across experimentally altered postnatal rearing conditions in a wild songbird
Source: Front Zool. 2018 Jun 19;15:25. doi: 10.1186/s12983-018-0272-y (PMC6006776; doi:10.1186/s12983-018-0272-y)
Supplement: Supplementary file 1 — Supplementary materials. Included are 2 supplementary tables (Tables S1 and S2). (DOCX 16 kb) [file 12983_2018_272_MOESM1_ESM.docx]

**SUPPLEMENTARY MATERIALS**

**The effect of pre-laying maternal immunization on offspring growth and immunity differs across experimentally altered postnatal rearing conditions in a wild songbird**

Rafał Martyka, Ewa B. Śliwińska, Mirosław Martyka, Mariusz Cichoń, Piotr Tryjanowski

**Tables**

**Table S1.** Results of general linear models that analyzed differences in body mass, clutch size, log-transformed LPS-specific Ab level and total Ab level in plasma between control and LPS-immunized females on the day of capture (PBS or LPS injection the day after first clutch completion). Group (control vs. immunized females) and year (to control for inter-season differences) were entered into all models as fixed factors.

| **Sources of variation** | **df** | **F** | **P** |
| --- | --- | --- | --- |
| Female body mass (g); N = 104 | | | |
| Year  Group | 1, 101  1, 101 | 15.00  0.00 | < 0.001  0.955 |
| Clutch size; N = 104 | | | |
| Year  Group | 1, 101  1, 101 | 1.37  0.06 | 0.245  0.815 |
| Log-transformed LPS-specific Ab level (mOD min^-1^); N = 101 | | | |
| Year  Group | 1, 98  1, 98 | 9.00  2.75 | 0.003  0.100 |
| Total Ab level (mOD min^-1^); N = 99 | | | |
| Year  Group | 1, 96  1, 96 | 22.45  1.39 | < 0.001  0.240 |

**Table S2.** Results of repeated-measures analysis of variance that examined differences in nestling body mass and brood sex ratio 2 days after hatching within a nest before and after cross-fostering. Nest state was a fixed factor that determined differences in measurements before and after swapping nestlings within a brood. Year was entered into both models as a fixed factor to control for inter-season differences.

| **Sources of variation** | **df** | **F** | **P** |
| --- | --- | --- | --- |
| Nestling body mass (g); N = 76 | | | |
| Year  Nest state  Year × nest state | 1, 72  1, 72  1, 72 | 5.36  1.07  0.08 | 0.026  0.308  0.773 |
| Brood sex ratio; N = 70 | | | |
| Year  Nest state  Year × nest state | 1, 66  1, 66  1, 66 | 0.32  0.23  0.12 | 0.575  0.631  0.730 |
