# Supplementary material for: The effect of pre-laying maternal immunization on offspring growth and immunity differs across experimentally altered postnatal rearing conditions in a wild songbird
Source: Front Zool. 2018 Jun 19;15:25. doi: 10.1186/s12983-018-0272-y (PMC6006776; doi:10.1186/s12983-018-0272-y)
Supplement: Supplementary file 2 — Supplementary materials. Included are detailed descriptions of ELISA assays performed to assess LPS-specific and total antibody levels. (DOCX 14 kb) [file 12983_2018_272_MOESM2_ESM.docx]

**SUPPLEMENTARY MATERIALS**

**The effect of pre-laying maternal immunization on offspring growth and immunity differs across experimentally altered postnatal rearing conditions in a wild songbird**

Rafał Martyka, Ewa B. Śliwińska, Mirosław Martyka, Mariusz Cichoń, Piotr Tryjanowski

**Immunological assays**

**Quantification of LPS-specific Ab levels in female and nestling plasma**

LPS-specific Abs were assessed on 96-well ELISA plates (MaxiSorp; Nunc). The plates were coated with 100 μl of LPS (Sigma, Cat. No. L-7261) at a concentration of 5 μg/ml suspended in carbonate buffer (0.15 M, pH 9.6) and incubated overnight at 4℃. The next day, after emptying the wells, the plates were blocked with 3% bovine serum albumin (BSA fraction V; Roche Diagnostics) suspended in 0.01 M phosphate*-*buffered saline (PBS; pH 7.2) for 2 h at room temperature, and then washed three times with 200 µl of 0.05% Tween 20 diluted in PBS. During incubation, the plasma samples from females and nestlings were diluted 1:50 in 1% BSA-PBS. After emptying and washing wells, 100 µl of the diluted plasma samples was added in duplicate to the wells on the plates. Importantly, pre- and post-immunization plasma samples of the same individual were placed on the same ELISA plate. Likewise, dilution series of the standard (1:50, 1:100, 1:200 and 1:400 dilutions) and negative control were added in duplicate on each plate. The standard was prepared from pooled plasma samples of all females and nestlings and served to assess the repeatability of measurements between and within plates. After adding samples to the wells, the plates were again incubated for 3 h at room temperature and then emptied and washed three times with 200 µl PBS-Tween 20. Subsequently, 100 µl of alkaline phosphatase-conjugated secondary Abs (Sigma, cat. no. A-9171) diluted 1:1,000 in 1% BSA-PBS was added, and the plates were incubated overnight at 4°C. Finally, the plates were emptied and washed three times with 200 µl PBS-Tween 20, and 100 µl of an alkaline phosphatase yellow liquid substrate (Sigma, Cat. No. P-7998) was added to all wells. The plates were immediately placed into a µQuant plate reader (Bio-Tek Instruments, Inc.) and read at 36-second intervals for 14 min with optical density (OD) measured at 405 nm. All measurements were reported as the slope of the substrate conversion (in 10^-3^ x OD; mOD) over time (mOD min^-1^). A steeper slope indicated a higher concentration of Abs in the sample. Final Ab concentration was calculated by averaging the duplicate values of each sample and thereafter by subtracting the mean value of the negative control from the measured and averaged values of Ab concentration to control for non-specific binding. The measurements of LPS-specific Abs were repeatable for samples collected in 2013 (between plates: r = 0.88, F_31, 32_ = 16.08, P < 0.001; within plates: r = 0.91, F_31, 32_ = 20.27, P < 0.001) and 2014 (between plates: r = 0.90, F_39, 40_ = 18.56, P < 0.001; within plates: r = 0.91, F_39, 40_ = 20.22, P < 0.001).

**Quantification of total Ab levels in female and nestling plasma**

Total Ab level was determined using a similar protocol that was applied to determine LPS-specific Abs. Many of the steps were the same, although there were some differences caused by the non-specific character of the quantified Abs. First, the plates were coated with anti-chicken Abs (Sigma, cat. no. C-6409) diluted 1:180 in carbonate buffer. Second, plasma samples of females and nestlings were diluted 1:5,000, and the standard was diluted 1:1,000, 1:4,000, 1:8,000 and 1:20,000. Third, alkaline phosphatase-conjugated secondary Abs were diluted 1:10,000. The remaining procedures were the same as for LPS-specific Ab measurements. The repeatability of measurements for total Abs was satisfactory for samples collected in 2013 (between plates: r = 0.91, F_31, 32_ = 21.71, P < 0.001; within plates: r = 0.95, F_31, 32_ = 36.35, P < 0.001) and 2014 (between plates: r = 0.97, F_39, 40_ = , P < 0.001; within plates r = 0.96, F_39, 40_ = 48.96, P < 0.001).
